# ﻿A new cave-dwelling species of *Trimma* (Teleostei, Gobiidae) from the Red Sea, with notes on Red Sea endemism in *Trimma* spp.

**DOI:** 10.3897/zookeys.1255.159341

**Published:** 2025-10-09

**Authors:** Viktor N. Peinemann, Lucia Pombo-Ayora, Walter A. Rich, Michael D. Fox, Darren J. Coker

**Affiliations:** 1 Marine Science Program, Division of Biological and Environmental Science and Engineering, King Abdullah University of Science and Technology, Thuwal 23955, Saudi Arabia; 2 Department of Marine Science, The University of Texas at Austin, Marine Science Institute, 78373 Port Aransas, Texas, USA; 3 KAUST Coral Restoration Initiative (KCRI), King Abdullah University of Science and Technology (KAUST), 23955 Thuwal, Saudi Arabia

**Keywords:** Biodiversity, biogeography, COI, cryptobenthic, Indian Ocean, phylogeny, taxonomy

## Abstract

A new species of the gobiid genus *Trimma* is described from the Farasan Banks in the southeastern Red Sea. The new species is characterized by having a predorsal midline with 7–8 scales, the fifth pelvic-fin ray unbranched, cheek and opercle scaleless, all pectoral-fin rays unbranched, and a dorsal fin VI + I,7, without elongate spines. In life, the species is bright yellow throughout, with a distinctive yellow-green longitudinal band in the central third of the dorsal fins. The new species inhabits caves on exposed offshore reefs at depths between 15 and 30 m where it occurs in small groups of up to 10 individuals. The new species appears to be sister to *T. winchi* from the western Indian Ocean. We also present a multilocus phylogeny (COI, 16S, Ptr, S7I1), including all known Red Sea *Trimma* and 21 non-Red Sea species, and an expanded supermatrix tree with 93 species to place Red Sea endemism in broader context. This brings the total number of *Trimma* species known from the Red Sea to 10, with eight appearing to be endemic to the region. The high proportion of endemism in the genus is noteworthy, even for the Red Sea, which has one of the highest proportions of endemic reef fishes in the Indo-Pacific. Moreover, K2P distances in the two widespread species suggest they may also represent cryptic endemic species, but further analyses are needed. The new species is currently known only from the Farasan Banks region despite extensive sampling along the Saudi Arabian Red Sea coast.

## ﻿Introduction

The gobiid genus *Trimma* Jordan & Seale, 1906 is one of the most speciose genera of coral reef-associated fishes in the Indo-Pacific, with over 110 valid described species. Commonly known as pygmygobies due to their small body size (typically <30 mm SL), members of this genus can be found on most Indo-Pacific coral reefs. Along with the similarly diverse and small-bodied *Eviota* Jenkins, 1903, they frequently represent one of the most abundant gobiids on hard substrates of coral reefs (Winterbottom 2019). Like *Eviota*, species of *Trimma* frequently show strong site fidelity and microhabitat specialization, likely contributing to their rapid diversification. New species are being discovered frequently, and molecular analyses suggest that many widespread species may represent complexes of multiple cryptic species with restricted geographic distributions (Winterbottom et al. 2020).

The Red Sea harbors a distinctive assemblage of *Trimma* species, with most being endemic to the region (Bogorodsky and Randall 2019). Most of these species show strong associations with specific reef zones or microhabitats. *Trimma flavicaudatum* (Goren, 1982) and *T. avidori* (Goren, 1978) often represent the dominant cryptobenthic species on many Red Sea reefs, in some cases with dozens of individuals per square meter (Coker et al. 2018). However, their niche overlap is limited, with typically only one of the species dominating in each habitat. Some species, including *T. barralli* Winterbottom, 1995 and *T. sheppardi* Winterbottom, 1984, primarily inhabit depths of at least 30 m, while others, such as *T. mendelssohni* (Goren, 1978), are common in the upper 10 m of the reef. Associations with caves, which are common for *Trimma* species throughout the Indo-Pacific, are also present in the Red Sea. Both *T. fishelsoni* Goren, 1985 and *T. nubarum* Winterbottom, Bogorodsky & Alpermann, 2023 live in cave-associated groups. The former prefers small groups of low density, while the latter can be observed in dense schools of upwards of 30 individuals, hovering above the substrate near the entrances of caves (pers. obs. of first author).

During biodiversity surveys of offshore reefs in the Farasan Banks region of the Saudi Arabian Red Sea, we discovered a distinctive, yellow-colored species of *Trimma* inhabiting cave systems at depths between 15–30 m. While often co-occurring with other cave-associated *Trimma* species, this taxon was typically restricted to areas slightly deeper inside the cave and more closely associated with the cave substrate. Herein we describe this new species based on morphological and molecular evidence and discuss its relationship to other members of the genus, particularly its apparent sister species *T. winchi*Winterbottom 1984 from the western Indian Ocean.

## ﻿Materials and methods

Morphological measurements and meristic counts follow standard protocols established by Richard Winterbottom to ensure comparability with previous work (Winterbottom 2019; Winterbottom et al. 2023 and references cited therein). Likewise, the format of the diagnosis and description follows the sequence of characters in the key to the genus (Winterbottom 2019) and other recent species descriptions (Winterbottom et al. 2023). We documented cephalic sensory papillae following established row naming conventions (Winterbottom 2011; Winterbottom and Erdmann 2015). Character ranges are reported as minimum – holotype – maximum, followed by the mean and number of specimens in parentheses. Values of the holotype are presented in bold.

Specimens were collected from cave habitats at depths of 15–30 m using clove oil anesthetic and hand nets. Live and fresh coloration was documented through photographs taken immediately after collection and during *in situ* observations. Ex-situ photographs were taken in an upright photo tank with a Nikon Z7 camera body, Nikon Z MC 105 mm f/2.8 macro lens, and a Godox MF12 Macro flash. To enhance visibility of key taxonomic features, particularly the cephalic sensory papillae, specimens were temporarily stained with cyanine blue 5R (acid blue 113).

For molecular analyses, we extracted genomic DNA from tissue samples using the Qiagen DNeasy Blood & Tissue Kit. A region of the cytochrome c oxidase subunit I (COI) gene was amplified using the primer sets FishF2 and FishR2 (Ward et al. 2005) and GOBYL6468 and GOBYH7696 (Thacker 2003). Resulting sequences were then cleaned and aligned in Geneious Prime 2025.0.3 to produce a 648-bp alignment. Pairwise genetic distances between species were calculated using MEGA11 using the Kimura 2-parameter (K2P) model (Kimura 1980; Tamura et al. 2021) on the COI alignment. We also amplified three additional loci from the same specimens: mitochondrial 16S rRNA using primers 16SarL and 16SbrH (Palumbi et al. 1991), nuclear S7 ribosomal protein intron 1 (S7I1) using primers S7I1F and S7I1R (Chow and Hazama 1998), and nuclear Protease III (Ptr) using primers PtrF2 and PtrR2 (Yamada et al. 2009). Ptr was included because it can help resolve deeper splits and has been used in the similarly speciose genus *Eviota* (Tornabene et al. 2013).

The final concatenated alignment consisted of 648 bp COI, 588 bp Ptr, 904 bp S7I1, and 608 bp 16S. The multi-locus dataset comprised specimens from our studies and collections, including all described Red Sea *Trimma*, 21 non-Red Sea *Trimma*, and two likely undescribed taxa: one Red Sea species similar to *T. anaima* Winterbottom, 2000 and one Madagascar species similar to *T. quadrimaculatum* Hoese, Bogorodsky & Mal, 2015. These specimens are currently being worked on by the authors, but they were included here due to their relevance to Red Sea endemism in *Trimma* spp.

A maximum-likelihood tree was inferred using W-IQ-TREE (Trifinopoulos et al. 2016) via the web interface at http://iqtree.cibiv.univie.ac.at/. Protein-coding genes (COI and Ptr) were partitioned by codon position; all loci were also partitioned by gene. The best-fit substitution model was automatically selected. Branch support was assessed with 1000 ultrafast bootstrap replicates. *Sueviota aethon* Peinemann, Pombo-Ayora & Tornabene, 2024 was included as an outgroup to root the tree. The phylogenetic tree was visualized using Interactive Tree Of Life (iTOL) v6 (Letunic and Bork 2024) and finalized in Adobe Illustrator 29.2.1.

In addition to the multi-locus dataset, we constructed a supermatrix that combines the full concatenated data for our specimens (COI, Ptr, S7I1, 16S) with COI-only terminals for *Trimma* species not represented in our collection. We queried the Barcode of Life Data System (BOLD) for all such species, selecting one representative COI sequence per species, with preference for longer sequences and for records identified by recognized experts. These public COI sequences were aligned to the COI partition of our concatenated dataset; the remaining three loci for those added taxa were coded as missing data. This preserves the higher signal of our multi-locus sampling while placing it in the context of the majority of described species of *Trimma*. Supp. material 1 lists, for each added species, the species name as used in the tree, the BOLD Process ID and, where available, the corresponding GenBank accession number, the name of the specimen identifier, and the collection locality. Supp. material 2 lists information for specimens in our collection used for the multi-locus dataset and the COI and 16S GenBank accession numbers generated for this study. The final supermatrix tree included all 33 *Trimma* species from our multi-locus dataset plus 60 additional COI-only species from BOLD, for a total of 93 *Trimma* species.

Type specimens have been catalogued and deposited in the ichthyology collection of the Royal Ontario Museum (ROM). All fieldwork was conducted under approval 20IAUCUC05 issued by the KAUST Institutional Animal Care and Use Committee (IACUC).

## ﻿Results

### ﻿ Trimma berumeni sp. nov.

Taxon classificationAnimaliaGobiiformesGobiidae

89DBA89D-CCF6-54A8-AD75-6C1DB12FC0C0

https://zoobank.org/9759ABBF-2807-4C55-9925-7598731B789B

**Figure 1.**
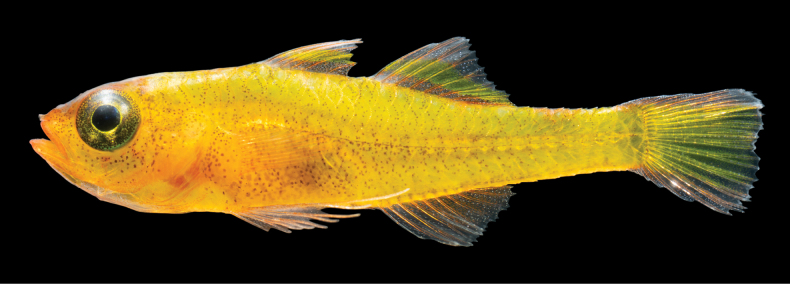
Freshly collected *Trimma berumeni* sp. nov., holotype, ROM 116871. Photograph: V. N. Peinemann.

**Figure 2.**
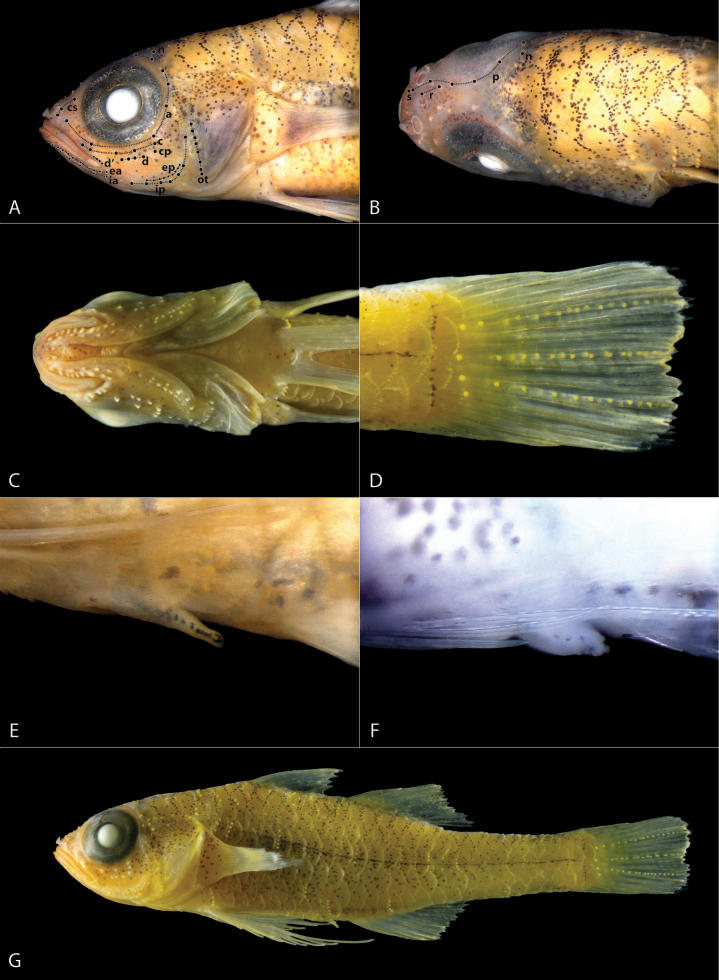
Close-up of key features of *Trimma berumeni* sp. nov., preserved in ethanol, ROM 116871 (A, B, C, D, E, G), ROM 116874 (F). A. Lateral view of head showing cephalic sensory papillae, specimen stained with cyanine blue, papillae highlighted with black dots, papilla rows connected by dotted lines and labeled accordingly; B. Dorsal view of head, showing cephalic sensory papillae and nasal apparatus, specimen stained with cyanine blue, papillae highlighted with black dots, papilla rows connected by dotted lines and labeled accordingly; C. Ventral view of head, showing cephalic sensory papillae, gill opening, and pelvic-fin base; D. Caudal fin with sensory papillae; E. Male urogenital papilla; F. Female urogenital papilla, specimen stained with cyanine blue; G. Lateral view of whole fish (preserved in 75% ethanol).

Figs 1, 2 Mikey’s golden pygmygoby

#### Type locality.

Saudi Arabia, Red Sea: Farasan Banks, 18.5041°N, 40.6606°E.

#### Type material.

***Holotype*** • ROM 116871, 13.6 mm SL male, Red Sea, Saudi Arabia, Farasan Banks, 18.5041°N, 40.6606°E, inside cave of exposed reef wall, 27 m depth, collected with clove oil and hand net, V. N. Peinemann, 5 June 2024.

***Paratypes*** • ROM 116872, *n* = 2, 13.3 mm SL male, Red Sea, Saudi Arabia, Farasan Banks, 18.5041°N, 40.6606°E, inside cave of exposed reef wall, 27 m and 15 m depth respectively, collected with holotype • ROM 116873, 13.8 mm SL male, Red Sea, Saudi Arabia, Farasan Banks, 18.8581°N, 40.3788°E, inside cave of exposed reef wall, 15 m depth, V. N. Peinemann, 6 June 2024 • ROM 116874, *n* = 3, 10.7 mm SL female, 10.9 mm SL male, and 10.7 mm SL male, Red Sea, Saudi Arabia, Farasan Banks, 19.8375°N, 39.9297°E, inside cave of exposed reef wall, 30 m depth, V. N. Peinemann, 19 May 2022.

#### Diagnosis.

A species of *Trimma* distinguished by the following combination of characters: predorsal midline with 7–8 scales; fifth pelvic-fin ray unbranched and 40–42% length of fourth ray; cheek and opercle scaleless; all pectoral-fin rays unbranched; body bright yellow throughout and without bars in life; dorsal fin VI + I,7; no elongate spines in dorsal fin; bony interorbital width 81–84% of pupil diameter; iris golden-yellow with melanophores except for golden ring around pupil and inverted golden triangle extending from ventral margin of pupil; fins translucent with yellow-green longitudinal band in central third of dorsal fins; a thin black midlateral line from above pectoral-fin base to caudal-fin base, visible in life and in preservative.

#### Description.

Based on holotype and 6 paratypes 10.7–13.8 mm SL. Dorsal fin VI + I,7, second spine longest but not elongate extending to base of second dorsal-fin spine when adpressed; all rays of second dorsal fin branched; second dorsal fin not elongate, reaching posteriorly 23–26–30 (27%, 7) of caudal peduncle length; anal fin I, 8, all rays branched; anal fin not elongate, reaching posteriorly 23–30 (27%, 7) of caudal peduncle length; pectoral-fin rays 14–15, all unbranched; pectoral-fin reaching posteriorly to vertical above urogenital papilla; pelvic fin I, 5, fifth ray unbranched and 40–42 (41%, 7) length of fourth ray, which reaches posteriorly to between base of first and third anal-fin ray, pelvic rays 1 to 4 with one branching point each; basal membrane approximately 9–10% of fourth pelvic-fin ray; no fraenum; caudal fin with 2 dorsal and 2–3 ventral segmented unbranched rays, and 6 dorsal and 5 ventral segmented branched rays; caudal fin with four vertically aligned papillae at base and three rows of 9–10 papillae each extending from the vertically aligned papillae to posterior margin of caudal fin (Fig. 2D); male urogenital papilla elongated and narrow (Fig. 2E; female urogenital papilla short and bulbous (Fig. 2F).

Lateral scales 24; anterior transverse scales 7–8, posterior transverse scales 7–8; cheek and opercle scaleless; 7–8 scales on predorsal midline; body scales ctenoid; circumpeduncular scales 12; scale rows in ventral midline between base of last anal-fin ray and first procurrent caudal-fin ray 7–8.

Gill opening extending anteroventrally to between middle and posterior third of pupil (Fig. 2C); anterior naris tubular reaching anteriorly across upper lip to posterior margin of lower lip; posterior naris oval and pore-like with raised rim, separated from bony front of orbit by 1.6–2 times its diameter (Fig. 2B); bony interorbital width 81–84% of pupil diameter (82%, 7); no dermal ridge on midline of nape extending anteriorly from origin of first dorsal fin.

Caudal peduncle depth as percentage of caudal peduncle length 30–41 (37.2%, 7); head length as percentage of SL 27–29–31 (29.3%, 7); horizontal eye diameter 40–45 (43.1%, 7); snout length 16–20 (18.9%, 7); and upper-jaw length 32–33 (32.6%, 7) as percentage of head length

Number of papillae in each row (Fig. 2A): a = 6 (4); b = 1–2 (5); c = 5 (5); d = 3–4 (5); d’ = 3–4 (5); p = 6 (5); e-anterior = 7–9 (5); e-posterior = 7–9 (5); i-anterior = 6–7 (5); i-posterior = 7 (5); cs” = 3 (5); ot = 7–8 (4); oi = 3–4 (4); u = 4 (3); cp = 1 (5); n = 1 (5); f = 2–3 (5).

***Color pattern, live*** (Fig. 1). Body bright yellow throughout. Head primarily yellow with orange coloration around the jaws, snout, interorbital region and below the ventral margin of the eye. Individuals can activate five translucent whitish bars along dorsal margin of body, from above the pectoral fin base to the caudal peduncle, sometimes visible *in situ*. Head and body with numerous small melanophores scattered throughout, running along scale pockets in the dorsal third of body, and scattered without patterns below the midlateral line. Specimens smaller than 11 mm SL with few melanophores on ventral half of body. A thin black midlateral line running along body from above pectoral-fin base to caudal-fin base. Iris golden-yellow, peppered with melanophores, except in a golden ring surrounding the pupil and an inverted golden triangle extending from ventral margin of the pupil. Dorsal fins translucent with dense red, white, and black chromatophores along basal third, a yellow-green longitudinal band running along central third, and sparse red, white, and black chromatophores along distal third. Anal fin mostly translucent with red, white, and black chromatophores, without yellow-green pigmentation. Dorsal and ventral fifths of caudal fin translucent with scattered red and white chromatophores, central section of caudal fin yellow to yellow-green. Pectoral fins and pelvic fins hyaline.

***Color pattern, preserved*** (Fig. 2G). Head and body primarily light yellow in large and recently preserved specimens, slowly fading to white in small specimens older than a year. Melanophores remain scattered throughout. A thin black midlateral line just under scales, extending from above pectoral-fin base to caudal-fin base. Iris densely packed with melanophores, appearing mostly black. Dorsal and anal fins hyaline with melanophores scattered in the posterior third of each fin, growing denser towards the posterior margin of the fin. Pectoral, pelvic, and caudal fins hyaline.

#### Etymology.

Named in honor of Michael L. Berumen in recognition of his substantial contributions to our understanding of the ecology and biodiversity of Red Sea coral reefs. Mikey’s golden pygmygoby is suggested as the common name.

#### Distribution and habitat.

*Trimma berumeni* sp. nov. inhabits caves of exposed offshore reefs, where it moves along the surface of cave roofs and walls in small groups of three to ten individuals (occasionally it is also solitary). It is typically found deep within caves, rarely seen within the first 80 cm of a cave entrance. The habitat is similar to that of its sister species, *Trimma winchi*, in the Seychelles (Ryan Daly pers. comm.).

Specimens were collected at depths between 15 and 30 m. While only two specimens were observed shallower than 20 m, the species is relatively common in caves at 30 m. Our collections and surveys were limited to 30 m, but it is likely that the species extends to greater depths. We observed this species exclusively on reefs with steep walls that extend well into the mesophotic.

**Figure 3.**
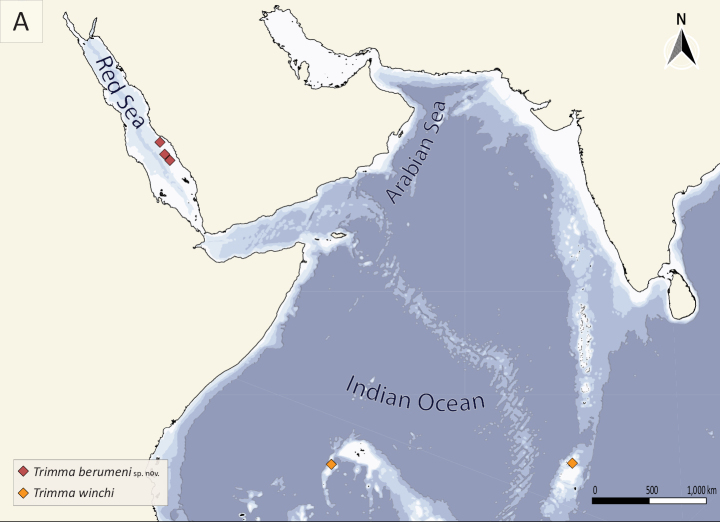
Map showing collection localities of *Trimma berumeni* sp. nov. (red diamonds; present study) and its sister species *T. winchi* (orange diamonds; Winterbottom 2019) in the Red Sea and Indian Ocean.

The species is present throughout much of the Farasan Banks in the southeastern Red Sea (Fig. 3). Despite extensive collections in similar habitats along the Saudi Arabian coastline north of the Farasan Banks, from Jeddah to Tiran, we have not observed this species anywhere but the Farasan Banks. It is possible that the species is endemic to the southern half of the Red Sea.

##### ﻿Comparisons

In the key to the genus (Winterbottom 2019), *T. berumeni* keys out to couplet 71 but does not match either of the two species in the couplet. *Trimma berumeni* differs from both in having 7 dorsal fin rays (vs 9 in *Trimma imaii* Suzuki & Senou, 2009 and 8 in *Trimma matsunoi* Suzuki Sakaue & Senou, 2012). It additionally differs from *T. imaii* in having a bony interorbital width wider than 80% of pupil width (vs 40% in *T. imaii*) and from *T. matsunoi* in having 7–8 predorsal midline scales (vs 6 in *T. matsunoi*). The general body, fin, and eye color is also clearly distinct in each species.

**Figure 4.**
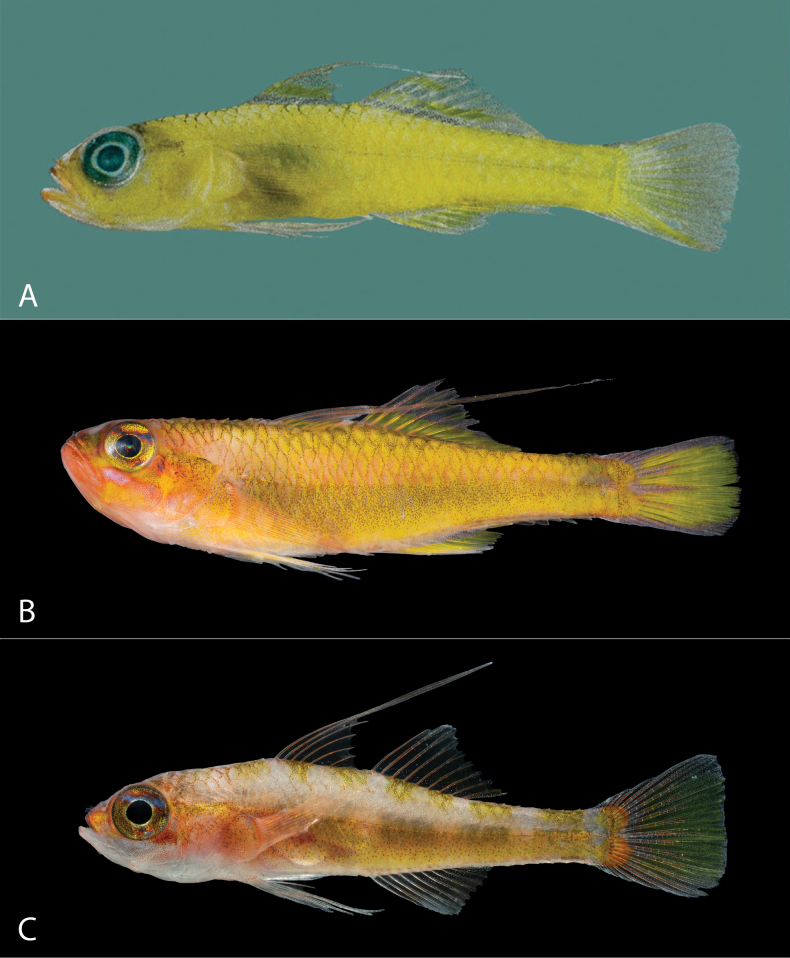
Comparison of species similar to *Trimma berumeni* sp. nov. in the Red Sea and Indian Ocean. A. *T. winchi*, holotype, ROM 41477, fresh, Salomon Atoll, Chagos Archipelago; B. *T. fishelsoni*, fresh, Straits of Tiran (northern Red Sea / Gulf of Aqaba), Saudi Arabia; C. *T. fishelsoni*, live, Obstruction Reef (Thuwal, central Red Sea), Saudi Arabia. Photographs: A. by R. Winterbottom; B, C. by V. N. Peinemann.

**Figure 5.**
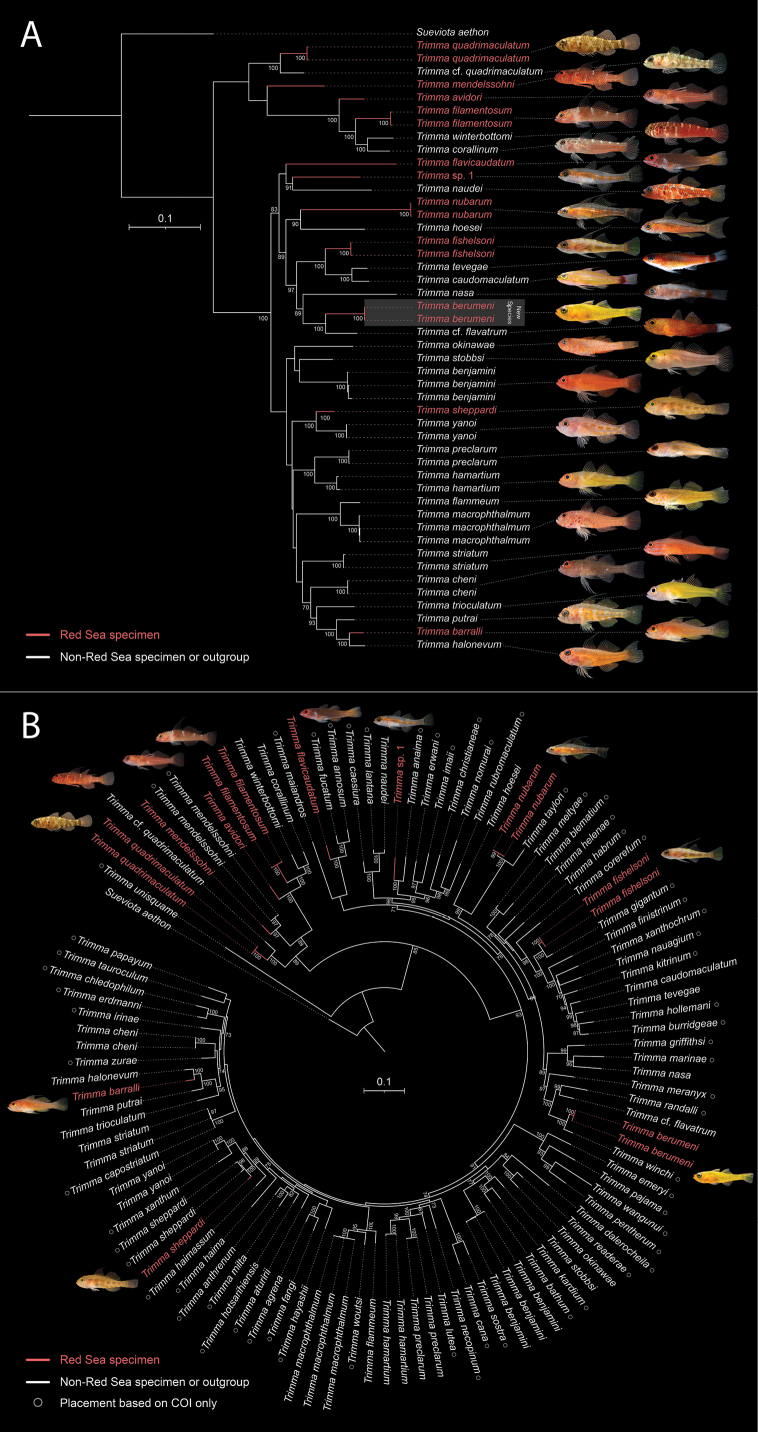
Phylogenetic placement of *Trimma berumeni* sp. nov. and Red Sea *Trimma* diversity. A. Maximum-likelihood tree from a concatenated alignment of COI, 16S, Ptr, and S7I1 for 33 species (all Red Sea *Trimma* plus selected Indo-Pacific relatives). Bootstrap values >70% are shown for internal nodes (1,000 ultrafast bootstrap replicates). *Sueviota aethon* is the outgroup. Scale bar represents 0.1 substitutions per site. Red branches and labels denote Red Sea specimens; among Red Sea *Trimma*, all species except *T. mendelssohni* and *T. sheppardi* are considered endemic species. Photographs correspond to the species listed at the tips, in order from top to bottom (photo credits: Red Sea *T. quadrimaculatum* S. V. Bogorodsky; all others V. N. Peinemann & L. Pombo-Ayora). Images are of the sequenced specimen or a conspecific from the same locality. B. Maximum-likelihood tree from a supermatrix (concatenated sequences from panel A + 60 additional species with COI only; total 93 species). Bootstrap values >70% are shown for internal nodes (1,000 ultrafast bootstrap replicates). Scale bar represents 0.1 substitutions per site. Red labels indicate Red Sea taxa; open circles mark tips placed using COI only. Photos are shown only for Red Sea species. Sequence sources, vouchers, and GenBank/BOLD accession numbers for all taxa shown are listed in Suppl. material 1 (COI-only taxa) and Supp. material 2 (multi-locus taxa).

In overall morphology, the *T. berumeni* is most similar to *T. winchi*, which is currently known from the Seychelles and the Chagos Archipelago (Fig. 4A). They share a uniform yellow body color, have a similar habitat, and a geographically close but non-overlapping distribution. The COI sequences of *T. berumeni* and *T. winchi* differ by a K2P distance of 6.4% (see Suppl. material 3 for K2P distances of Red Sea *Trimma* and sister taxa in Fig. 5B). Several traits can be used to separate the two species. These are summarized in Table 1. Notably, the two Chagos (type locality) male specimens of *T. winchi* have an elongate dorsal spine, which is absent in Ryan Daly’s photographs of *T. winchi* from the Seychelles. It has not been evaluated whether this represents a distinct species or intraspecific variation. In Table 1 we follow the original description in listing dorsal-spine elongation for *T. winchi*, but the variation warrants further investigation.

**Table 1.** Comparison of key characters between *Trimma berumeni* sp. nov. and *T. winchi*. Data for *T. winchi* based on Winterbottom (1984), Winterbottom (2019), and examination of a photograph of holotype.

	Trimma berumeni sp. nov.	Trimma winchi
Geographic range	Farasan Banks, southern Red Sea	Seychelles and Chagos Archipelago
Dorsal fin	VI + I,7	VI + I,8
Elongate dorsal spines	None; second spine longest reaching to base of second dorsal-fin spine when adpressed	Second spine elongate, reaching posteriorly just beyond base of last dorsal-fin ray when adpressed (absent in Seychelles specimens)
Second dorsal-fin length	Last ray not elongate, reaching posteriorly 23–30% of caudal peduncle length	Last ray elongate, reaching posteriorly two-thirds of caudal peduncle length
Anal-fin length	Last ray not elongate, reaching posteriorly 23–30% of caudal peduncle length	Last ray elongate, reaching posteriorly two-thirds of caudal peduncle length
Dorsal-fin color (alive)	Black longitudinal basal stripe composed of large melanophores with scattered iridocytes; single yellow longitudinal band above it; distal third hyaline	Black longitudinal basal stripe composed of large melanophores with scattered iridocytes; a central yellow longitudinal band, then a narrow hyaline band; distal quarter yellow
Anal-fin color (alive)	No yellow pigmentation, mostly translucent with red, white, and black chromatophores	Mostly yellow, with basal band of melanophores and iridocytes
Caudal-fin color (alive)	Yellow with translucent dorsal and ventral margins	Yellow throughout with distal margin of melanophores

Within the Red Sea, *T. fishelsoni* bears some similarity to *T. berumeni*. Both species have a primarily yellow body color, a yellow longitudinal band along the dorsal fins, and a primarily yellow caudal fin. Both species also have an overlapping distribution in the central to southern Red Sea and are associated with caves. *Trimma berumeni* can be distinguished from *T. fishelsoni* by having no elongate dorsal spines (vs second spine elongate and filamentous in *T. fishelsoni*), 7–8 predorsal midline scales (vs 9–14 in *T. fishelsoni*), scaleless cheek and operculum (vs scaled in *T. fishelsoni*), a basal membrane connecting the fifth pelvic fin rays (vs absent in *T. fishelsoni*). The live color of *T. berumeni* differs by the body being bright yellow throughout (vs yellow with a white to purple stripe above midlateral line in *T. fishelsoni*), head yellow throughout (vs primarily pale to pink in lower jaw and ventrally of eye in *T. fishelsoni*), no longitudinal yellow band on anal fin (vs usually present in *T. fishelsoni*), no blotches at caudal fin base (vs two large vertically aligned yellow-orange blotches at caudal fin base in *T. fishelsoni*), and no lines across iris (vs oblique blue line with orange-red margins across upper edge of pupil in *T. fishelsoni*).

Bogorodsky et al. (2016) extended the known range of *T. fishelsoni* from the northern Red Sea to the Farasan Banks, while noting some morphological differences between the populations. The southern population has no branching pectoral fin rays while northern ones have some. Southern populations lack the four saddle-like dorsal blotches described for Gulf of Aqaba specimens (more apparent in preservative) by Goren (1984). Gulf of Aqaba specimens (Fig. 4B) also appear to have a yellow patch at the posterior margin of the jaw that is absent or less distinctive in southern populations. We have collected specimens from the central Red Sea with a mostly pale dorsal body color and 5 or 7 dusky yellow dorsal blotches (either a single large or two smaller blotches are present under each dorsal fin) that were apparent in live, freshly dead, and preserved individuals (Fig. 4C). These individuals have no branching pectoral fin rays and a much fainter yellow color on the anal fin. While further analyses and additional specimens are needed to determine the nature of this variation between *T. fishelsoni* populations, the differences to *T. berumeni* discussed above are consistent across populations.

## ﻿Discussion

The Red Sea is a peripheral sea characterized by high levels of endemism shaped by its geographic isolation and distinct environmental conditions. The Bab-el-Mandeb Strait limits gene flow between the Red Sea and the Indian Ocean, while upwelling systems along the eastern Somali and southern Arabian coasts create ecological barriers that further restrict dispersal. These factors have contributed to an exceptionally high level of endemism in Red Sea fishes (DiBattista et al. 2016a, 2016b; Bogorodsky and Randall 2019). New putative endemic species continue to be discovered frequently, including in the same regions and cave habitats as the new *Trimma berumeni* (Winterbottom et al. 2023; Nunes Peinemann et al. 2024).

With the description of *Trimma berumeni*, the number of described *Trimma* species recorded from the Red Sea rises to 10, of which eight are currently considered endemic species. Two species, *T. mendelssohni* and *T. sheppardi*, are currently recognized as widespread but exhibit relatively deep genetic divergence between their Red Sea and non-Red Sea populations (4–10% K2P distance). While further study is needed, these divergences suggest that the Red Sea lineages may represent cryptic endemic species rather than truly widespread taxa. In comparison, the K2P distance between *T. berumeni* and *T. winchi* is 6.4%. Geographically restricted haplogroups and potential cryptic speciation are well documented in *Trimma* spp. (Winterbottom et al. 2014, 2020, 2023). For completeness, we also included two putatively undescribed taxa, T. cf. quadrimaculatum (Madagascar) and a Red Sea *T.* sp. 1 similar to *T. anaima*, which each form Red Sea/non-Red Sea sister pairs in our trees (Fig. 5): the former to the Red Sea *T. quadrimaculatum* lineage (13.5% COIK2P) and the latter to *T. anaima* outside the Red Sea (15.6% COIK2P). They are included solely for context and are not treated further in this paper.

Our 93-species maximum-likelihood tree places *T. berumeni* in a well-supported sister pairing with *Trimma winchi* (Fig. 5B). Although the placement of *T. winchi* is based solely on the COI gene (and it was not included in the multi-locus analysis; Fig. 5A), this relationship is further supported by similarities in their morphology and habitat preferences. The two species maintain distinct but adjacent geographic ranges, with *T. berumeni* found in the central to southern Red Sea and *T. winchi* in the western Indian Ocean. More broadly, our analyses suggest that most Red Sea *Trimma* have a sister taxon located outside the Red Sea (Fig. 5). This pattern is consistent with multiple colonization events rather than a single evolutionary radiation within the basin. Such phylogenetic overdispersion, where endemic species are scattered across various Indo-Pacific lineages, mirrors patterns observed in other Red Sea reef fish families (DiBattista et al. 2016b).

Despite the high number of endemics, *in situ* diversification within the Red Sea appears to be uncommon in fishes, although there is some evidence of the Red Sea exporting diversity to the Indian Ocean (Bowen et al. 2013; DiBattista et al. 2013). In *Trimma*, COIK2P distances (Supp. material 3) suggest Red Sea endemism could be as high as 100%, yet our analyses provide no strong evidence for diversification occurring within the basin. The distribution of Red Sea taxa across otherwise Indo-Pacific lineages is parsimoniously explained by repeated, independent colonizations of the Red Sea; while an older in-basin event followed by speciation outside the Red Sea cannot be excluded, the current evidence does not require it.

Interpretation of our phylogenetic results warrants caution given data limitations. Although Fig. 5A incorporates multiple loci and Fig. 5B broadens taxon coverage, most non-Red Sea species are represented by COI alone. Because the supermatrix is anchored on COI, deeper placements of COI-only terminals should be regarded as provisional. Accordingly, we use the expanded tree primarily to frame Red Sea/non-Red Sea sister pairings rather than to infer deeper relationships among Indo-Pacific lineages. Denser taxon sampling with additional loci may provide valuable insights into the origin of Red Sea *Trimma* and *Trimma* evolution overall.

## Supplementary Material

XML Treatment for Trimma berumeni

## ﻿Additional information

### Use of AI

No use of AI was reported.

### Author ORCIDs

Viktor N. Peinemann https://orcid.org/0000-0002-7060-397X

Lucía Pombo-Ayora https://orcid.org/0000-0003-0316-0280

Walter A. Rich https://orcid.org/0000-0002-1304-5676

Michael D. Fox https://orcid.org/0000-0002-5004-1025

Darren J. Coker https://orcid.org/0000-0002-5099-0984

### Data availability

All of the data that support the findings of this study are available in the main text or Supplementary Information.

## Supplementary materials

Supplementary material 1 COI-only taxa included in the expanded 93-species phylogenetic dataset Data type xlsx Explanation note For each species, the table lists the species name as used in the tree, the BOLD Process ID, and where available the corresponding GenBank accession number, the name of the specimen identifier, and the collection locality. https://binary.pensoft.net/file/1434664This dataset is made available under the Open Database License (http://opendatacommons.org/licenses/odbl/1.0/). The Open Database License (ODbL) is a license agreement intended to allow users to freely share, modify, and use this Dataset while maintaining this same freedom for others, provided that the original source and author(s) are credited.Viktor N. Peinemann, Lucia Pombo-Ayora, Walter A. Rich, Michael D. Fox, Darren J. Coker

Supplementary material 2 Specimens included in the multi-locus dataset generated for this study and used in the phylogenetic analyses, with associated voucher numbers, collection localities, and GenBank accession numbers for COI and 16S Data type xlsx https://binary.pensoft.net/file/1434665This dataset is made available under the Open Database License (http://opendatacommons.org/licenses/odbl/1.0/). The Open Database License (ODbL) is a license agreement intended to allow users to freely share, modify, and use this Dataset while maintaining this same freedom for others, provided that the original source and author(s) are credited.Viktor N. Peinemann, Lucia Pombo-Ayora, Walter A. Rich, Michael D. Fox, Darren J. Coker

Supplementary material 3 Pairwise genetic distances (Kimura 2-parameter) between COI sequences of *Trimma* species from the Red Sea and Indo-Pacific Data type xlsx Explanation note The new species, *T. berumeni*, is highlighted in bold. Values shown as percentages. GenBank accession numbers and collection localities are provided for each sequence. Cells are color-coded from yellow (high divergence) to green (low divergence) to aid visualization. https://binary.pensoft.net/file/1434666This dataset is made available under the Open Database License (http://opendatacommons.org/licenses/odbl/1.0/). The Open Database License (ODbL) is a license agreement intended to allow users to freely share, modify, and use this Dataset while maintaining this same freedom for others, provided that the original source and author(s) are credited.Viktor N. Peinemann, Lucia Pombo-Ayora, Walter A. Rich, Michael D. Fox, Darren J. Coker
